# *CTNND1* is involved in germline predisposition to early-onset gastric cancer by affecting cell-to-cell interactions

**DOI:** 10.1007/s10120-024-01504-7

**Published:** 2024-05-25

**Authors:** Cristina Herrera-Pariente, Laia Bonjoch, Jenifer Muñoz, Guerau Fernàndez, Yasmin Soares de Lima, Romesa Mahmood, Miriam Cuatrecasas, Teresa Ocaña, Sandra Lopez-Prades, Gemma Llargués-Sistac, Xavier Domínguez-Rovira, Joan Llach, Irina Luzko, Marcos Díaz-Gay, Conxi Lazaro, Joan Brunet, Carmen Castillo-Manzano, María Asunción García-González, Angel Lanas, Marta Carrillo, Raquel Hernández San Gil, Enrique Quintero, Nuria Sala, Gemma Llort, Lara Aguilera, Laura Carot, Pilar Diez-Redondo, Rodrigo Jover, Teresa Ramon y Cajal, Joaquín Cubiella, Antoni Castells, Francesc Balaguer, Luis Bujanda, Sergi Castellví-Bel, Leticia Moreira

**Affiliations:** 1 Gastroenterology, Fundació de Recerca Clínic Barcelona-Institut d’Investigacions Biomèdiques August Pi I Sunyer (FRCB-IDIBAPS), CIBEREHD, Universitat de Barcelona, Hospital Clínic, Villarroel 170, 08036 Barcelona, Spain; 2 Hospital Sant Joan de Déu, CIBERER, 08950 Barcelona, Spain; 3 Pathology, Hospital Clínic, FRCB-IDIBAPS, CIBEREHD, 08036 Barcelona, Spain; 4 Department of Cellular and Molecular Medicine and Department of Bioengineering and Moores Cancer Center, UC San Diego, La Jolla, San Diego, CA 92093 USA; 5 Hereditary Cancer Program, Catalan Institute of Oncology, IDIBELL, CIBERONC, 08908 Barcelona, Spain; 6 Hereditary Cancer Program, Catalan Institute of Oncology, IDIBGI, 17190 Girona, Spain; 7 Hereditary Cancer Program, Catalan Institute of Oncology, 08916 Badalona, Spain; 8 Instituto de Investigación Sanitaria Aragón, Instituto Aragonés de Ciencias de La Salud, CIBEREHD, 50009 Zaragoza, Spain; 9 Gastroenterology, Hospital Clínico Universitario de Zaragoza, Instituto de Investigación Sanitaria Aragón, Universidad de Zaragoza, CIBEREHD, 50009 Zaragoza, Spain; 10 Gastroenterology, Centro de Investigación Biomédica de Canarias (CIBICAN), Hospital Universitario de Canarias, Instituto Universitario de Tecnologías Biomédicas (ITB), Universidad de La Laguna, 38320 Santa Cruz de Tenerife, Spain; 11 Department of Medical Oncology, Hospital Universitario de Canarias, 38320 Tenerife, Spain; 12 Unit of Nutrition and Cancer, Translational Research Laboratory, Catalan Institute of Oncology (ICO) and Bellvitge Biomedical Research Institute (IDIBELL), 08908 Barcelona, Spain; 13 Medical Oncology, Parc Taulí University Hospital, 08208 Sabadell, Spain; 14 Gastroenterology, Vall d’Hebron Research Institute, 08035 Barcelona, Spain; 15 Gastroenterology, Hospital del Mar, 08003 Barcelona, Spain; 16 Hospital Universitario Río Hortega, 47012 Valladolid, Spain; 17 Gastroenterology, Departamento de Medicina Clínica, Hospital General Universitario Dr. Balmis, Instituto de Investigación Sanitaria ISABIAL, Universidad Miguel Hernández, 03010 Alicante, Spain; 18 Medical Oncology, Santa Creu I Sant Pau Hospital, 08041 Barcelona, Spain; 19 Gastroenterology, Complexo Hospitalario de Ourense, CIBEREHD, 32005 Ourense, Spain; 20 Department of Hepatology and Gastroenterology, Centro de Investigación Biomédica en Red de Enfermedades Hepáticas y Digestivas (CIBERehd), Biodonostia Health Research Institute - Donostia University Hospital, Universidad del País Vasco (UPV/EHU), 20014 San Sebastián, Spain; 21 Facultat de Medicina i Ciències de la Salut, Universitat de Barcelona (UB), Barcelona, Spain

**Keywords:** Stomach neoplasms, Delta catenin, Early-onset disease, Genetic predisposition to disease, Cell adhesion

## Abstract

**Background:**

*CDH1* and *CTNNA1* remain as the main genes for hereditary gastric cancer. However, they only explain a small fraction of gastric cancer cases with suspected inherited basis. In this study, we aimed to identify new hereditary genes for early-onset gastric cancer patients (EOGC; < 50 years old).

**Methods:**

After germline exome sequencing in 20 EOGC patients and replication of relevant findings by gene-panel sequencing in an independent cohort of 152 patients, *CTNND1* stood out as an interesting candidate gene, since its protein product (p120ctn) directly interacts with E-cadherin. We proceeded with functional characterization by generating two knockout *CTNND1* cellular models by gene editing and introducing the detected genetic variants using a lentiviral delivery system. We assessed β-catenin and E-cadherin levels, cell detachment, as well as E-cadherin localization and cell-to-cell interaction by spheroid modeling.

**Results:**

Three *CTNND1* germline variants [c.28_29delinsCT, p.(Ala10Leu); c.1105C > T, p.(Pro369Ser); c.1537A > G, p.(Asn513Asp)] were identified in our EOGC cohorts. Cells encoding *CTNND1* variants displayed altered E-cadherin levels and intercellular interactions. In addition, the p.(Pro369Ser) variant, located in a key region in the E-cadherin/p120ctn binding domain, showed E-cadherin mislocalization.

**Conclusions:**

Defects in *CTNND1* could be involved in germline predisposition to gastric cancer by altering E-cadherin and, consequently, cell-to-cell interactions. In the present study, *CTNND1* germline variants explained 2% (3/172) of the cases, although further studies in larger external cohorts are needed.

**Supplementary Information:**

The online version contains supplementary material available at 10.1007/s10120-024-01504-7.

## Introduction

Gastric cancer (GC) is the fifth most common cancer with more than 1,000,000 new cases in 2020. Both environmental and genetic factors are involved in GC susceptibility. Up to 10% of all GC cases display familial aggregation and up to 5% are caused by germline predisposition [1]. Some of these cases are explained by genetic variants in the known hereditary GC genes. Among them, *CDH1* (encoding E-cadherin) [2] and *CTNNA1* (encoding α-E-catenin) [2, 3], and *APC* promoter 1B pathogenic variants [4], have been related to hereditary syndromes with high risk mainly of GC (hereditary diffuse GC syndrome, or HDGC, and gastric adenocarcinoma and proximal polyposis of the stomach, respectively). In addition, there are other genes involved in other hereditary cancer syndromes that increase the probability to develop GC besides other tumors. They include Lynch syndrome (DNA mismatch repair genes), hereditary breast-ovarian cancer syndrome (*BRCA1/2, PALB2*), as well as familial adenomatous polyposis (*APC/MUTYH*), juvenile polyposis (*SMAD4/BMPR1A*), Li-Fraumeni (*TP53*), Peutz-Jeghers (*STK11)* and Cowden syndromes (*PTEN*) [1].

Despite this already known GC germline predisposition, there are still some families with GC aggregation without an identified hereditary cause. There is a similar situation for early-onset GC patients (EOGC), a subgroup of GC patients characterized by a diagnosis at 50 years or earlier and clinicopathologically different from conventional GC [5]. However, 10% of them have a positive family history, and only some cases are explained by other hereditary cancer syndromes [5]. Therefore, there is a need to identify new germline predisposition genes that could explain these unsolved cases, either with familial aggregation or early-onset presentation. In addition, it is known that inherited factors have a strong role in tumor development in EOGC patients. In fact, an increased incidence of GC in young people (< 50 y.o.) has been described [6], highlighting even more this need. Therefore, studying this subgroup of patients is a useful approach to decipher their germline background.

During the last years, next-generation sequencing has become a useful tool to identify new candidate genes for EOGC germline predisposition [2, 7–9]. Nevertheless, this technology generates a huge number of genetic variants that, at the end, need to be further validated by functional studies to establish a clear and strong association between the candidate germline predisposition factor and the disease.

We previously proposed some candidate genes after exome sequencing (WES) in a discovery cohort of 20 EOGC patients [10]. In the present study, we aimed to identify novel causal genes for EOGC germline predisposition. Accordingly, we replicated WES results by targeted gene-panel sequencing and functionally validated three rare variants detected in a candidate gene.

## Materials and methods

### Patients

#### Clinical characteristics

A discovery cohort of 20 EOGC patients was previously studied [10]. For replication purposes, 152 unrelated EOGC patients were recruited in 12 different Spanish hospitals. All of them developed GC before the age of 50 and no previous germline pathogenic variants were identified in the already known hereditary GC genes. In this replication cohort, 47.2% of the patients were women and the median age at GC diagnosis was 42.0 y.o. Oncologic, personal and family history characteristics are summarised in Table 1.

**Table 1:** Clinical characteristics of the replication cohort (*n* = 152)

Age at cancer diagnosis; median (IQR)	42·0 (37·0–46·0)
Gender (women); number (%)	68/144 (47·2)
Personal history of other neoplasm, number (%)	0
Family history of GC (*n* = 139)	
(FDR, SDR, TDR), number (%)	51 (36·7)
Age at cancer diagnosis; median (IQR)	59·0 (46·0–69·0)
Family history of other tumors (*n* = 134)	
(FDR, SDR, TDR), number (%)	
Breast	37 (27·6)
Colorectal	35 (26·1)
Lung	10 (7·5)
Ovarian	8 (6·0)
Laryngeal	7 (5·2)
Prostate	5 (3·7)
Neck/head	4 (3·0)
Melanoma	4 (3·0)
Thyroid	4 (3·0)
Liver	4 (3·0)
Brain	3 (2·2)
Bladder	2 (1·5)
Uterus	2 (1·5)
Pancreatic	2 (1·5)
Endometrial	2 (1·5)
Glioblastoma	1 (0·7)
Phyllodes	1 (0·7)
Tumor stage (*n* = 82), number (%)	
I/II	26 (31·7)
III/IV	56 (68·3)
GC histology (*n* = 113), number (%)	
Diffuse	80 (70·8)
Intestinal	26 (23·0)
Mixed	6 (5·3)
Medullary	1 (0·9)

*IQR* interquartile range, *GC* gastric cancer, *FDR* first-degree relative, *SDR* second-degree relatives, *TDR* third-degree relatives, (*n* = x) represents the number of patients with available information

### Immunohistochemistry and loss of heterozygosity

Immunostainings and loss of heterozygosity assessment were performed when tumor sample was available. See online Supplemental material for more details.

### Variant identification

#### DNA extraction

Germline DNA samples were extracted using the QIAamp DNA Blood kit (Qiagen, Hilden, Germany) following the manufacturer’s instructions.

#### Gene panel sequencing

After WES in a discovery cohort of 20 EOGC patients [10], a custom gene panel was designed. This panel had 38 genes, including 24 genes potentially associated with hereditary GC (based on our previous WES analysis) and 14 external genes already associated with a higher risk of GC. See online Supplemental material for further details.

#### Gene panel analysis

Raw-sequencing data were aligned to the hg19 human genome using the Burrows-Wheeler Aligner v.0.7.15 (BWA-MEM). Then, variant calling was conducted using Genome Analysis Toolkit (GATK). Variant annotation was performed using SnpEff software (https://pcingola.github.io/SnpEff/). Afterward, variants were filtered by an in-house R pipeline, as previously described [10]. Briefly, this pipeline selects rare (allele frequency ≤ 0.1%), nonsynonymous and truncating genetic variants, and missense genetic variants with at least three out of six pathogenicity prediction tools [11–16] in the case of missense variants. Afterward, those genes with variants in both discovery and replication cohorts were further considered.

#### Variant validation

Integrative Genome Viewer (IGV; http://software.broadinstitute.org/software/igv/) and Sanger sequencing (Eurofins Genomics, Luxembourg) were performed for a final validation of the identified genetic variants. Primers are listed in Table S1.

### Cellular model development for variant characterization

#### Cell lines

Human gastric cancer NCI-N87 (Cat No. CRL-5822), human immortalized retinal hTERT RPE-1 (Cat No. CRL-4000) and human kidney HEK293T (Cat No. CRL-3216) cell lines were obtained from American Type Culture Collection (ATCC, Manassas, VA). NCI-N87 cell line was cultured with RPMI-1640 medium (Gibco, Waltham, MA) and hTERT RPE-1 and HEK293T cells were cultured with DMEM medium (Gibco, Waltham, MA), both supplemented with 10% fetal bovine serum. All cell lines were cultured in standard growth conditions (37 ºC, 5% CO_2_) and tested for mycoplasma contamination with the Mycoplasma Gel Detection kit from Biotools (Madrid, Spain).

For information regarding *CTNND1* (NM_001085458.1) loss-of-function model generation and variant reintroduction, protein extraction, Western blot and Detachment assay, see online Supplementary material.

### Functional characterization of genetic variants

#### Wnt pathway: β-catenin and E-cadherin expression.

Β-catenin and E-cadherin protein expression was assessed by Western Blot. The primary antibodies used were anti-β-catenin (clone C10A8, Cat No. 8480, Cell Signaling) and anti-E-cadherin (clone 4A2, Cat No. ab231303, Abcam), in combination with anti-GAPDH (clone 14C10, Cat No. 2118, Cell signaling) for normalization purposes.

#### Generation of spheroids

Spheroids were generated using Nunclon Sphera treated U-Shaped-Bottom 96-Well plates (Cat No. 174925, ThermoFisher, Waltham, MA). Briefly, 200 uL containing 1,000 cells and 5,000 cells were seeded per well for NCI-N87 and hTERT RPE models, respectively. After 4 days of spheroid formation, pictures were taken with an Olympus IX51 phase-contrast microscopy at 4 × magnification to assess their size, shape and integrity.

#### Imaging analysis of spheroids

All images were analyzed using the open-source software Fiji and a macro designed by Ivanov et al., [17] adapted to properly characterize NCI-N87 and hTERT RPE spheroids. Briefly, this macro converts the picture to black and white, applies a thresholding algorithm, cleans the artifacts, fills holes in the spheroid and generates a file with all the measured size parameters and shape descriptors (incl. area, circularity, roundness, solidity, aspect ratio) selected in the analyze particles command.

#### Immunofluorescence of spheroids

NCI-N87 spheroids were seeded at a concentration of 5,000 cells/well for 4 days, and hTERT RPE spheroids at a concentration of 5,000 cells/well during 24 h. Protocol was adapted from Gaskell et al. [18]. Spheroids were transferred to Eppendorf tubes, washed three times with PBS and fixed with 4% formaldehyde for 1 h at room temperature (RT). Then, spheroids were washed again and permeabilized with 0.5% Triton X-100 in PBS with 0.05% Tween20 overnight at 4 ºC. Then, they were blocked with 0.1% Triton X-100 with 20% normal goat serum (NGS) in PBS for 2 h at RT. Spheroids were blotted overnight at 4 ºC with primary antibodies anti-p120ctn and anti-E-cadherin diluted 1:200 in 0.1% triton X-100 with 10% NGS in PBS. The following day, spheroids were washed three times with 0.1% triton X-100 in PBS (1 h/wash) and blotted overnight at 4 ºC with the secondary antibody previously diluted 1:1000 in 0.1% triton X-100 with 10% NGS in PBS. Secondary antibodies were anti-mouse Alexa fluor 488 (Cat No A28175, ThermoFisher, Waltham, MA) and anti-rabbit Alexa fluor 594 (Cat No. A-11012, ThermoFisher, Waltham, MA). The next day, spheroids were washed three times with 0.1% triton X-100 in PBS (30 min/wash). After DAPI nuclear staining for 20 min, two quick washes with PBS were done. Then, spheroids were mounted with Ibidi Mounting medium (Cat No. 50001, Ibidi, Fitchburg, WI) in a microscope slide and stored at 4 ºC for subsequent observation. LEICA DM 2500 confocal microscope (10 × and 63 × oil objectives) was used for immunoflourescence (IF) detection.

## Results

### Variant identification

Besides WES data from 20 EOGC patients of the discovery cohort [10], custom gene-panel sequencing data was available from 152 EOGC patients of the replication cohort. After variant prioritisation, only rare, nonsynonymous/truncating or missense variants with at least three out of six pathogenic predictions were further considered from both the discovery and the replication cohort. Among them, those located on the catenin delta-1 (*CTNND1*) gene stood out. Its protein product, catenin delta-1, also known as p120ctn, is involved in cell-to-cell adhesion and signal transduction. Specifically, it directly interacts with E-cadherin, a well-known predisposing gene for hereditary diffuse GC, and is considered the main inhibitor of E-cadherin endocytosis [19].

In the discovery cohort [10], the WES-data prioritization detected one genetic variant in *CTNND1* [c.28_29delinsCT, p.(Ala10Leu)] in patient 9. This genetic variant was classified as pathogenic by six prediction tools and the patient, who developed GC at 41 years, displayed diffuse histology. In the replication cohort, two additional *CTNND1* genetic variants were found. Both c.1105C > T, p.(Pro369Ser) and c.1537A > G, p.(Asn513Asp) variants were classified as damaging by three out of six pathogenic prediction tools. These variants were identified in patient 154 (onset at 44 years) with intestinal histology and patient 187 (onset at 45 years) with diffuse histology, respectively. Sanger sequencing confirmed the three genetic variants (Fig. S1a-c). None of the three patients were aware of familial GC aggregation. Any case of other type of cancer was identified in the family members of patient 9 and patient 154, while family history of patient 187 showed a sister with brain tumor and both maternal uncle and grandfather with prostate cancer. Variant information is listed in Table S2.

Loss of heterozygosity of the wild-type allele was analyzed in the tumors of c.28_29delinsCT, p.(Ala10Leu) and c.1537A > G, p.(Asn513Asp) carriers, but it was only detected in the former (Fig. S1a, S1c). DNA from the tumor of the c.1105C > T, p.(Pro369Ser) variant carrier was not available. Immunohistochemical staining of p120ctn and E-cadherin proteins were performed in tumors of c.1105C > T, p.(Pro369Ser) and c.1537A > G, p.(Asn513Asp) variant carriers (Fig. S2). Loss of p120ctn expression was found in c.1105C > T, p.(Pro369Ser) tumor, while E-cadherin expression was not altered. In c.1537A > G, p.(Asn513Asp) variant carrier, some tumor cells show focal and weak membrane staining.

### Functional characterization of *CTNND1* loss-of-function

To further validate the impact of *CTNND1* variants and their link to EOGC germline predisposition, this gene was targeted using CRISPR-Cas9 technology in two different cell lines: NCI-N87, a gastric cell line expressing both p120ctn and E-cadherin, and hTERT RPE-1, an immortalized cell line which expresses p120ctn but N-cadherin instead of E-cadherin [20] (Fig. S3). One knockout clone of each cell line (termed hereafter NCI KO10 and RPE KO7, respectively) were selected. *CTNND1* gene editing was confirmed by Sanger sequencing and no expression was found at protein level in both clones (Fig. S3a-d). In addition, NCI KO10 showed evident morphological changes after *CTNND1* inactivation (Fig. S3e).

**Fig. 1:**
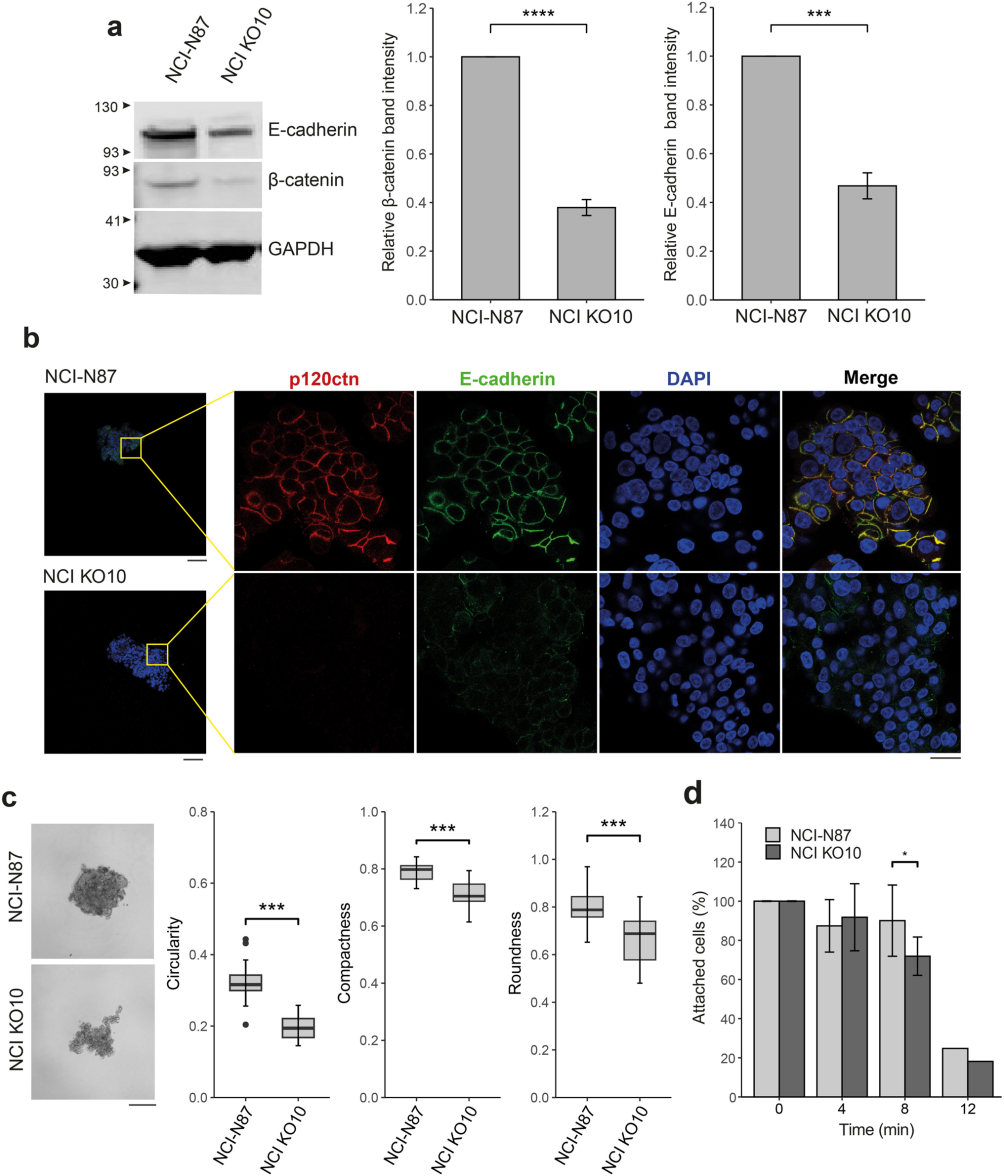
Functional characterization of the *CTNND1*-depleted NCI-N87 cell model by CRISPR/Cas9. **a** Quantification of relative protein levels of E-cadherin and β-catenin by Western blot. GAPDH was used as internal control. Representative blot of n = 4 (mean ± SD; Welch’s t-test, ^∗∗∗^*P* < 0.001). **b** Immunofluorescent staining of spheroids (NCI-N87 and NCI KO10) using p120ctn and E-cadherin antibodies. CRISPR-mediated *CTNND1* editing resulted in p120ctn complete depletion and reduced E-cadherin expression. Wide-field microscopy images (left panel) were captured using a 10 × objective (Scale bar = 100 µm). A small area is shown at higher magnification (right panel, 63 × oil objective, scale bar = 20 µm). **c** Representative pictures of NCI-N87 and NCI KO10 spheroids. Scale bar = 200 µm. Circularity, compactness and roundness index decreased after p120ctn deletion. Spheroids were generated at least in quintuplicate and the experiment was repeated 3 times (n = 3; mean ± SD; Welch’s t-test, ^∗∗∗^*P* < 0.001). **d** Detachment time-course assay. Data represent the percentage of remaining attached cells after being treated with trypsin at different timepoints (n = 9, mean ± SD; Welch’s t-test, ^∗^*P* < 0.05)

Then, we evaluated how *CTNND1* depletion could alter different cell functions. p120ctn directly interacts with the cytoplasmatic domain (juxtamembrane domain, JMD) of E-cadherin. Both are members of adherens junctions where β-catenin, a key element of Wnt pathway, is also involved. We evaluated protein expression of both E-cadherin and β-catenin, finding a significant lower expression of both proteins in NCI KO10 (Fig. 1a). On the contrary, although there seems to be a reduction, no significant differences regarding β-catenin levels were found in RPE KO7 (Fig. S4a). Since hTERT RPE-1 cell line expresses N-cadherin instead of E-cadherin, E-cadherin protein levels were not evaluated.

It is well known that p120ctn is involved in cell-to-cell adhesion by controlling cadherin turnover. It prevents E-cadherin internalization by hampering its interaction with the ubiquitin ligase Hakai or the endocytic machinery [21–23]. Since *CTNND1*-depleted cells showed both morphological and E-cadherin alterations, hampered cell-to-cell interaction was suspected. Bearing this in mind, we engineered NCI KO10 and RPE KO7 spheroids, a useful technique to study cell-to-cell interactions [24], and evaluated E-cadherin and p120ctn localization by IF. p120ctn was properly located at the membrane in both cell lines, although hTERT RPE-1 cells displayed a dotted p120ctn pattern along the membrane (Fig. 1b and Fig. S4b). Regarding E-cadherin, we observed a notable decrease of its membrane levels in NCI KO10 spheroids (Fig. 1b), in concordance with what was detected by Western Blot. Nevertheless, there was not a clear E-cadherin vesicular internalization pattern in these cells. As expected, E-cadherin was not detected in hTERT RPE-1 cells (Fig. S4b).

To further characterize *CTNND1* knock-outs and changes in cell-to-cell adhesion, three-dimensional spheroid modeling and cell detachment assays were performed. Both NCI KO10 and RPE KO7 spheroids were less compact than their wild-type counterparts. Whereas NCI KO10 spheroids resembled loose cell aggregates, RPE KO7 showed cell layers surrounding the spheroid itself as a consequence of p120ctn inactivation. The analysis of spheroid images showed a significant decrease in all shape descriptors, including circularity, compactness and roundness (Fig. 1c and Fig. S4c), which reflects weak intercellular interactions [25]. These results were supported by the trypsin-based cell detachment assay. Monolayer cultures of both *CTNND1* knockout models showed a significantly impaired cell-to-cell adhesion capacity after 8 or 3 min of treatment in NCI KO10 and RPE KO7, respectively (Fig. 1d and Fig. S4d). These time points were chosen for further experiments.

Altogether, these results showed that the inactivation of *CTNND1* in NCI-N87 and hTERT RPE-1 cell lines reduced E-cadherin and β-catenin expression, with a significant decrease observed in NCI KO10-N87, and compromised cell-to-cell adhesion.

### Functional characterization of *CTNND1* germline variants

The three genetic variants identified in both discovery and replication cohorts were studied to evaluate their functional effect. The variants p.(Ala10Leu), p.(Pro369Ser) and p.(Asn513Asp) were generated by site-directed mutagenesis (Fig. S3f) and reintroduced into both NCI KO10 and RPE KO7 using a lentiviral delivery system. In addition, a wild-type version of *CTNND1* was reintroduced as a control to restore the original phenotype. The doxycycline dose of 100 ng/uL was selected and a successful gene expression for all the tested conditions was achieved (Fig. S3g).

**Fig. 2:**
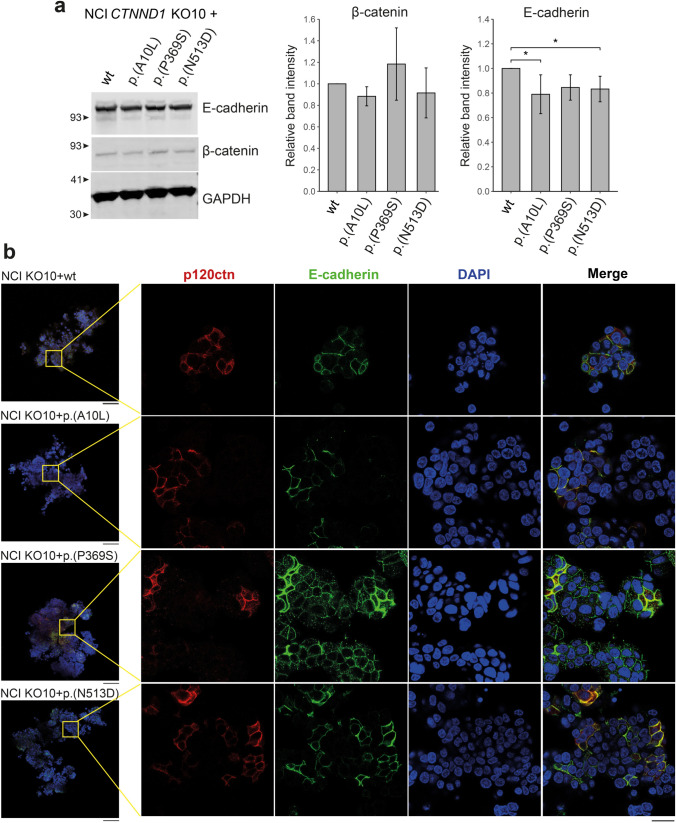
*CTNND1* variants compromise E-cadherin levels and its membrane localization. **a** Quantification of relative protein levels of E-cadherin and β-catenin by Western blot. GAPDH was used as internal control. Representative blot of *n* = 4 (mean ± SD; analysis of variance with LSD post hoc test, ^*^P < 0.05). **b** Immunofluorescent staining of spheroids expressing p120ctn variants. The vesicular internalization pattern of E-cadherin can be detected in cells expressing *CTNND1* p.(Pro369Ser). Wide-field microscopy images (left panel) were captured using a 10 × objective (Scale bar = 100 µm). A small area is shown at higher magnification (right panel, 63 × oil objective, scale bar = 20 µm). Protein annotation is in its short form

After establishing the model, functional assays were performed. First, we studied the cadherin/catenin complex. E-cadherin and β-catenin protein levels were evaluated in NCI KO10 cells by re-expressing the wild-type sequence or each genetic variant. No significant differences were found regarding β-catenin levels. However, p.(Ala10Leu) and p.(Asn513Asp) variants showed a significant decrease in E-cadherin expression (Fig. 2a). Then, we evaluated p120ctn and E-cadherin localization in NCI spheroids. All p120ctn genetic variants were properly located at the membrane. Interestingly, the p.(Pro369Ser) variant displayed a dotted pattern of E-cadherin (Fig. 2b), suggesting its mislocalization. Therefore, either E-cadherin expression or localization was impaired in cells expressing any of the *CTNND1* genetic variants.

**Fig. 3:**
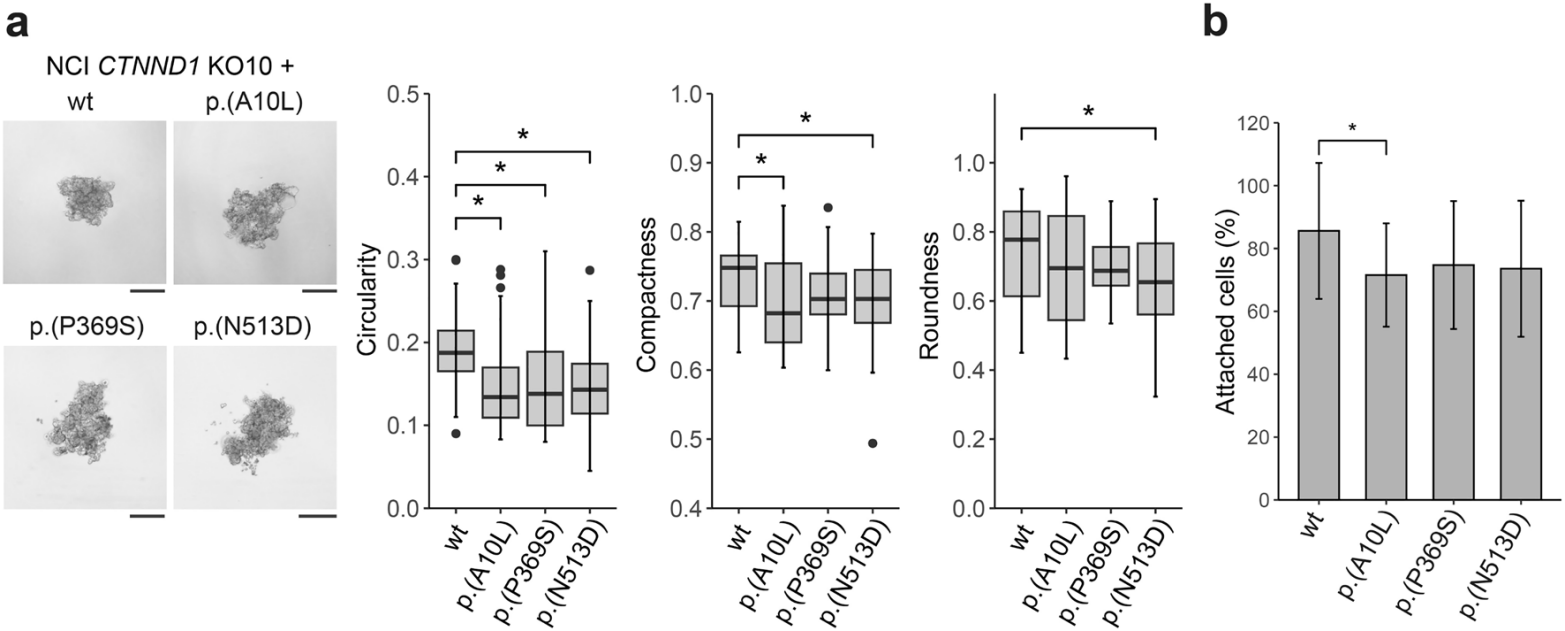
*CTNND1* variants affect cell-to-cell interactions. **a** Representative pictures of spheroids expressing each *CTNND1* variant or its wild-type counterpart. Circularity, compactness and roundness index are represented. Spheroids were generated at least in quintuplicate and the experiment was repeated 3 times (*n* = 3; mean ± SD; analysis of variance with LSD post hoc test, ^∗^*P* < 0.05). **b** Detachment assay. Data represent the percentage of remaining attached cells after being treated with trypsin for 8 min (*n* = 9, mean ± SD; analysis of variance with LSD post hoc test, ^∗^*P* < 0.05). Protein annotation is in its short form

The effect of p120ctn variants on cell-to-cell adhesion was further investigated through spheroid modeling and detachment assays. In terms of spheroid formation, all genetic variants promoted an irregular shape and altered cell assembly to some extent. All variants caused a significant decrease in spheroid circularity when compared to the wild-type counterpart. In addition, spheroid compactness was significantly reduced for p.(Ala10Leu) and p.(Asn513Asp) variants, with the latter also showing altered spheroid’s roundness (Fig. 3a). Regarding the detachment assay, all three variants led to reduced cell-to-cell attachment, but a significant difference was only observed for p.(Ala10Leu) (Fig. 3b).

This functional characterization was replicated in RPE KO7 cells after wild-type and genetic variants’ reintroduction. Regarding β-catenin levels, although there was a decreasing tendency, no significant differences were found (Fig. S5a). p120ctn expression was located mainly along the membrane but also in the cytoplasm, showing the same vesicular dotted pattern previously seen in hTERT RPE-1 parental cells (Fig. S5b). The assessment of cell-to-cell adhesion revealed that cells expressing *CTNND1* genetic variants showed a decrease only in spheroid circularity, although not statistically significant (Fig. S6a). In addition, no significant differences were observed in the detachment assay (Fig. S6b).

In our EOGC cohorts, *CTNND1* functionally pathogenic variants explained around 2% of cases (3/172). To further test the association between *CTNND1* and EOGC, we performed a gene-based burden test comparing rare, protein-disruption variants found in our EOGC cohort and those variants found in control subjects (gnomAD v.2.1.1). To select pathogenic variants, rare and loss-of-function variants were selected in this control dataset and nine cases (with splicing or protein-altering variants) were found (9/125,739; 0.007%). This confirms an enrichment for rare, pathogenic *CTNND1* variants in our EOGC cohort (χ^2^ = 543.88; *p* value = 0.00001).

## Discussion

This study builds on the previous fundings obtained by our group [10]. We have conducted a replication in a cohort of 152 EOGC patients by gene panel sequencing. Combining these results [10], we identified three patients with three heterozygous missense variants in the *CTNND1* gene. To validate its role in EOGC germline predisposition, cellular models using CRISPR-Cas9 technology were produced and then, functional assays were developed.

*CTNND1* is a member of the Armadillo protein family. Its protein product, p120ctn, has a crucial role in maintaining cell-to-cell adhesion at adherens junctions, regulating the activation of small RhoGTPases in the cytoplasm and modulating nuclear transcription. Specifically, it directly interacts within the JMD of E-cadherin and controls E-cadherin turnover by hampering Hakai and endocytic machinery interaction [21–23].

**Fig. 4:**
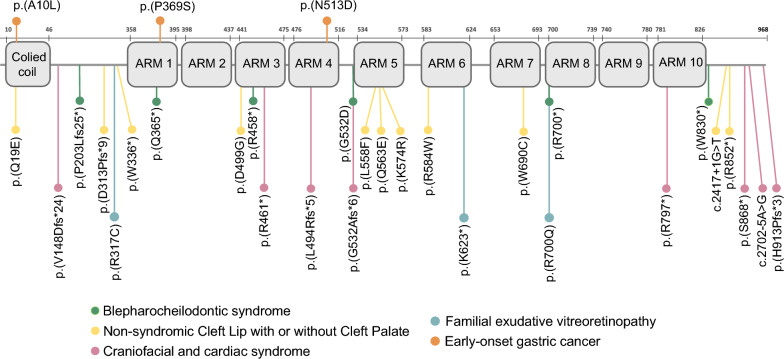
Schematic representation of p120ctn variants within the functional domains of the protein. *ARM* Armadillo domain

*CTNND1* has previously been associated with some hereditary syndromes with birth defects, including blepharocheilodontic syndrome [26, 27], nonsyndromic cleft lip with or without palate (CLP) [28] and a craniofacial and cardiac syndrome [29]. Recently, *CTNND1* germline variants have been also linked with familial exudative vitreoretinopathy [30]. GC was not diagnosed in any of these patients. Blepharocheilodontic syndrome and nonsyndromic cleft lip/palate have also been related to *CDH1* germline variants [26–28]. Interestingly, even some HDGC patients with *CDH1* germline variants display CLP too [31–33]. In addition, familial exudative vitreoretinopathy syndrome has also been linked to *CTNNA1* inherited variants [34]. Both *CDH1* and *CTNNA1* are well-established genes associated with HDGC predisposition [2, 35], supporting that *CTNND1* germline variants could be involved not only in other hereditary syndromes [26–30] but also in EOGC predisposition. In fact, although *CTNND1* was not found altered in a small cohorts of HDGC [36], a recent study has found up to six variants of unknown significance in *CTNND1* in a cohort of 141 GC patients with diffuse and mixed histology [37]. This finding increases the evidence of the possible involvement of *CTNND1* in germline predisposition to EOGC. In addition, it seems that frameshift variants are more frequent in the other syndromes rather than in EOGC, where missense variants could be more common (Fig. 4). This fact might explain phenotypical differences among these diseases.

In the present study, we demonstrated that *CTNND1* loss-of-function disrupts the cadherin/catenin complex and alters cell-to-cell interactions. In *CTNND1-*depleted cells, E-cadherin and β-catenin levels were downregulated, as previously reported [30]. Without p120ctn, Hakai or endocytic machineries interact with E-cadherin causing its internalization and degradation [21, 22] and therefore, β-catenin is also released into the cytoplasm and degraded [38]. Ultimately, this led to morphological changes, reduced adhesion capacity and an impaired spheroid formation. Similar results were obtained with the RPE model. Nevertheless, this cell line expresses preferably N-cadherin instead of E-cadherin [20]. E-cadherin interacts with shorter p120ctn isoforms, more frequent in epidermis, tongue and palate epithelia or in ducts of excretory glands, while N-cadherin associates with longer isoforms, more common in pigment epithelium of retina, among other cell types [39]. In addition, p120ctn was not properly located at the membrane of RPE cells and presented a dotted, vesicular staining pattern, suggesting that, at least, part of p120ctn (probably the shorter isoform that binds preferably to E-cadherin) was in the cytoplasm or being internalized [40]. Despite the lack of E-cadherin expression, p120ctn inactivation also altered cell adhesion ability and spheroid formation in RPE cells, since N-cadherin also mediates adherens junctions and needs to be bound to p120ctn to be properly delivered to the membrane [41].

After depicting the effect of *CTNND1* loss-of-function, we functionally validated the three different *CTNND1* missense genetic variants and assessed cadherin/catenin protein status and cell-to-cell interactions. Interestingly, the level of functional evidence supporting *CTNND1* malfunction can vary depending on the variant’s location within p120ctn functional domains: p.(Ala10Leu) is located at the coiled-coil domain in N-terminal end, while p.(Pro369Ser) and p.(Asn513Asp) variants are located in ARM1 and ARM4 domains, respectively (Fig. 4). Of particular note is the different effects observed either over E-cadherin expression or localization. It has been reported that the stability of E-cadherin at the adherens junctions is dependent on an equilibrium of cadherin retention at the membrane, driven by p120ctn, and cadherin internalization, induced by either the clathrin endocytic machinery or the Hakai-dependent ubiquitination and subsequent degradation [23]. Since the endocytic or ubiquitination fate of E-cadherin seems to be associated with different p120ctn binding regions, the specific location of p120ctn variants may influence or favor one mechanism over another.

Cells expressing either p.(Ala10Leu) or p.(Asn513Asp) variants showed decreased E-cadherin levels. The coiled-coil domain (aa 10–46) has been described as indispensable for E-cadherin stabilization [42]. In addition, the first 27 amino acids of p120ctn are necessary for a proper deliver of the cadherin/catenin complex to the membrane [43]. In our study, we also found impaired cell adhesion and intercellular interactions associated with the p.(Ala10Leu) variant. Given these observations, this variant, located in an important region of p120ctn, seems to be involved in GC predisposition. On the other hand, the p.(Asn513Asp) variant is located in ARM4, close to the dynamic E-cadherin binding region and to essential aminoacid positions (Asn478 and Asn536) [23]. The alteration of this intermediate-to-weak protein interaction could explain the lower levels of E-cadherin due to degradation. In addition, p.(Asn513Asp) cells showed significant morphological changes in spheroid formation but not differences in the two-dimensional cell detachment assay. This fact highlights spheroid modeling as a more suitable approach to study intercellular interactions than monolayer cellular attachment to tissue culture plates.

Regarding p.(Pro369Ser) variant, we did not observe any alteration in E-cadherin protein levels, but we detected an altered localization pattern by IF, suggesting clathrin-mediated E-cadherin internalization. The p.(Pro369Ser) variant is located in the ARM1 domain, within a hydrophobic pocket necessary for the static interaction between p120ctn and the JMD of E-cadherin. In addition, some specific amino acid positions (Pro366 and Val371), located very close to our variant, have been identified as key components for this binding [23]. This fact could support that alterations in or around these important positions could alter p120ctn-E-cadherin binding and result in E-cadherin vesicular internalization. In addition, cells expressing p.(Pro369Ser) variant exhibited an altered spheroid formation capacity, which collectively reinforces the alteration of the cadherin/catenin complex and consequently, intercellular junctions. Surprisingly, the p.(Pro369Ser) carrier showed GC with tubular (intestinal) histology, despite E-cadherin alterations have been classically linked to poorly cohesive (diffuse) histology [44]. GC tubular (intestinal) histology is more common in older patients [5], although EOGC patients can also present tubular (intestinal) histology [45]. At the somatic level, E-cadherin has been found altered in both histological types [46], confirming the role of this protein in both types. It is possible that the lack of differences in E-cadherin protein levels could be a potential compensatory mechanism in response to internalization. In fact, E-cadherin expression was not altered in the patient’s tumor, while loss of p120ctn expression was confirmed. In this context, further studies will be needed to elucidate the mechanism behind this finding.

Using hTERT RPE cells we have been able to characterize the effect of *CTNND1* variants beyond their interaction with E-cadherin. In this model, p120ctn complete depletion (RPE KO7) impaired cell adhesion and spheroid formation, whereas p120ctn variants did not affect cell-to-cell interactions. Although N- and E-cadherin are both type I cadherins, p120ctn has been suggested to bind more tightly to E-cadherin than to N-cadherin [47]. Therefore, it could be hypothesized that p120ctn missense variants associated with EOGC predisposition might impair cell-to-cell interactions mainly affecting E-cadherin, but not other cadherin family members, while complete p120ctn depletion could hamper adherens junctions in which N-cadherin is also involved.

The discovery of *CTNND1* as a new hereditary gastric cancer gene would permit identifying high-risk individuals and establishing preventive measures, early diagnosis, and personalized treatments. This finding could guide future studies in other external cohorts without a known germline cause to further identified new genetic variants in this gene that could help to improve disease management.

However, the present study has several limitations. First, histological sections and tumoral DNA were not available for all patients, which would have been valuable for a comprehensive understanding of the mutational status of *CTNND1* in all of them. In addition, although none of the patients showed familial aggregation of GC, analyzing the germline status of *CTNND1* in their relatives could have provided insight into confirming the hereditary pattern of *CTNND1* variants or its penetrance. In addition, the effect on cell-growth due to the alteration of cell-to-cell interactions by *CTNND1* variants has not been tested. Lastly, it is important to note that further analysis of *CTNND1* germline variants in international cohorts is necessary to accumulate evidence and elucidate the extent of our findings.

**Fig. 5:**
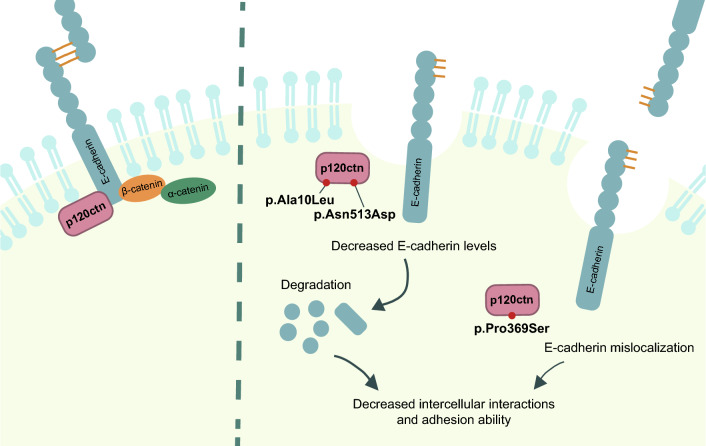
Schematic representation of the proposed molecular mechanism of pathogenicity for *CTNND1* germline variants. *CTNND1* germline variants affect p120ctn-E-cadherin binding and compromise cell-to-cell interactions, which could lead to GC predisposition

In summary, alterations in p120ctn could affect E-cadherin stability, promoting its internalization and/or degradation, and consequently reducing intercellular interactions (Fig. 5). Accordingly, our findings suggest that pathogenic variants in *CTNND1* could be involved in EOGC germline predisposition. In our EOGC cohorts, *CTNND1* germline predisposition explained around 2% of cases (3/172). Replication in additional cohorts and functional validation of the already-found variants in other external cohorts will be needed to further characterize its frequency and role in EOGC and GC.

### Supplementary Information

Below is the link to the electronic supplementary material.

Supplementary file1 (PDF 24847 KB)

## Data Availability

The sequencing dataset supporting the current study remain confidential because are currently under study but are available from the corresponding author on request.

## References

[1] Lott PC, Carvajal-Carmona LG (2018). Resolving gastric cancer aetiology: an update in genetic predisposition. Lancet Gastroenterol Hepatol.

[2] Hansford S, Kaurah P, Li-Chang H, Woo M, Senz J, Pinheiro H (2015). Hereditary diffuse gastric cancer syndrome: CDH1 mutations and beyond. JAMA Oncol.

[3] Clark DF, Michalski ST, Tondon R, Nehoray B, Ebrahimzadeh J, Hughes SK (2020). Loss-of-function variants in CTNNA1 detected on multigene panel testing in individuals with gastric or breast cancer. Genet Med.

[4] Li J, Woods SL, Healey S, Beesley J, Chen X, Lee JS (2016). Point mutations in exon 1B of APC reveal gastric adenocarcinoma and proximal polyposis of the stomach as a familial adenomatous polyposis variant. Am J Hum Genet.

[5] Milne AN, Offerhaus GJA (2010). Early-onset gastric cancer: Learning lessons from the young. World J Gastrointest Oncol.

[6] Arnold M, Park JY, Camargo MC, Lunet N, Forman D, Soerjomataram I (2020). Is gastric cancer becoming a rare disease? A global assessment of predicted incidence trends to 2035. Gut.

[7] Sahasrabudhe R, Lott P, Bohorquez M, Toal T, Estrada AP, Suarez JJ (2017). Germline mutations in PALB2, BRCA1, and RAD51C, which regulate DNA recombination repair, in patients with gastric cancer. Gastroenterology.

[8] Vogelaar IP, Van Der Post RS, Van Krieken JHJM, Spruijt L, Van Zelst-Stams WAG, Kets CM (2017). Unraveling genetic predisposition to familial or early onset gastric cancer using germline whole-exome sequencing. Eur J Hum Genet.

[9] Weren RDA, Van Der Post RS, Vogelaar IP, Han Van Krieken J, Spruijt L, Lubinski J (2018). Role of germline aberrations affecting CTNNA1, MAP3K6 and MYD88 in gastric cancer susceptibility. J Med Genet.

[10] Herrera-Pariente C, Capó-García R, Díaz-Gay M, Carballal S, Muñoz J, Llach J (2021). Identification of new genes involved in germline predisposition to early-onset gastric cancer. Int J Mol Sci.

[11] Zeng P, Zhao Y, Liu J, Liu L, Zhang L, Wang T (2014). Likelihood ratio tests in rare variant detection for continuous phenotypes. Ann Hum Genet.

[12] Sim N-L, Kumar P, Hu J, Henikoff S, Schneider G, Ng PC (2012). SIFT web server: predicting effects of amino acid substitutions on proteins. Nucleic Acids Res.

[13] Adzhubei IA, Schmidt S, Peshkin L, Ramensky VE, Gerasimova A, Bork P (2010). A method and server for predicting damaging missense mutations. Nat Methods.

[14] Schwarz JM, Cooper DN, Schuelke M, Seelow D (2014). Mutationtaster2: mutation prediction for the deep-sequencing age. Nat Methods.

[15] Kircher M, Witten DM, Jain P, O’Roak BJ, Cooper GM, Shendure J (2014). A general framework for estimating the relative pathogenicity of human genetic variants. Nat Gene.

[16] Siepel A, Pollard KS, Haussler D, Apostolico A, Guerra C, Istrail S, Pevzner PA, Waterman M (2006). New Methods for Detecting Lineage-Specific Selection. Res Comput Mol Biol.

[17] Ivanov DP, Parker TL, Walker DA, Alexander C, Ashford MB, Gellert PR (2014). Multiplexing spheroid volume, resazurin and acid phosphatase viability assays for high-throughput screening of tumour spheroids and stem cell neurospheres. PLoS ONE.

[18] Gaskell H, Sharma P, Colley HE, Murdoch C, Williams DP, Webb SD (2016). Characterization of a functional C3A liver spheroid model. Toxicol Res.

[19] Figueiredo J, Söderberg O, Simões-Correia J, Grannas K, Suriano G, Seruca R (2013). The importance of E-cadherin binding partners to evaluate the pathogenicity of E-cadherin missense mutations associated to HDGC. Eur J Hum Genet.

[20] Youn Y-H, Jeehee H, Burke JM (2006). Cell Phenotype in Normal Epithelial Cell Lines with High Endogenous N-Cadherin: Comparison of RPE to an MDCK Subclone. Invest Ophthalmol Vis Sci.

[21] Fujita Y, Krause G, Scheffner M, Zechner D, Leddy HEM, Behrens J (2002). Hakai, a c-Cbl-like protein, ubiquitinates and induces endocytosis of the E-cadherin complex. Nat Cell Biol.

[22] Miyashita Y, Ozawa M (2007). Increased Internalization of p120-uncoupled E-cadherin and a requirement for a dileucine motif in the cytoplasmic domain for endocytosis of the protein. J Biol Chem.

[23] Ishiyama N, Lee SH, Liu S, Li GY, Smith MJ, Reichardt LF (2010). Dynamic and static interactions between p120 catenin and E-cadherin regulate the stability of cell-cell adhesion. Cell.

[24] Habanjar O, Diab-Assaf M, Caldefie-Chezet F, Delort L (2021). 3D Cell Culture Systems: Tumor Application, Advantages, and Disadvantages. Int J Mol Sci.

[25] Leung BM, Cai Lesher-Perez S, Matsuoka T, Moraes C, Takayama S (2015). Media additives to promote spheroid circularity and compactness in hanging drop platform. Biomater Sci.

[26] Ghoumid J, Stichelbout M, Jourdain A-S, Frenois F, Lejeune-Dumoulin S, Alex-Cordier M-P (2017). Blepharocheilodontic syndrome is a CDH1 pathway–related disorder due to mutations in CDH1 and CTNND1. Genet Med.

[27] Kievit A, Tessadori F, Douben H, Jordens I, Maurice M, Hoogeboom J (2018). Variants in members of the cadherin–catenin complex, CDH1 and CTNND1, cause blepharocheilodontic syndrome. Eur J Hum Genet.

[28] Cox LL, Cox TC, Moreno Uribe LM, Zhu Y, Richter CT, Nidey N (2018). Mutations in the epithelial cadherin-p120-catenin complex cause mendelian non-syndromic cleft lip with or without cleft palate. Am J Hum Genet.

[29] Alharatani R, Ververi A, Beleza-Meireles A, Ji W, Mis E, Patterson QT (2020). Novel truncating mutations in CTNND1 cause a dominant craniofacial and cardiac syndrome. Hum Mol Genet.

[30] Yang M, Li S, Huang L, Zhao R, Dai E, Jiang X (2022). CTNND1 variants cause familial exudative vitreoretinopathy through the Wnt/cadherin axis. JCI Insight.

[31] Kluijt I, Siemerink EJM, Ausems MGEM, van Os TAM, de Jong D, Simões-Correia J (2012). CDH1-related hereditary diffuse gastric cancer syndrome: clinical variations and implications for counseling. Int J Cancer.

[32] Benusiglio PR, Caron O, Consolino E, Duvillard P, Coulet F, Blayau M (2013). Cleft lip, cleft palate, hereditary diffuse gastric cancer and germline mutations in CDH1. Int J Cancer.

[33] Obermair F, Rammer M, Burghofer J, Malli T, Schossig A, Wimmer K (2019). Cleft lip/palate and hereditary diffuse gastric cancer: report of a family harboring a CDH1 c.687 + 1G > a germline mutation and review of the literature. Fam Cancer.

[34] Zhu X, Yang M, Zhao P, Li S, Zhang L, Huang L (2021). Catenin α 1 mutations cause familial exudative vitreoretinopathy by overactivating Norrin/β-catenin signaling. J Clin Invest.

[35] Majewski IJ, Kluijt I, Cats A, Scerri TS, De Jong D, Kluin RJC (2013). An α-E-catenin (CTNNA1) mutation in hereditary diffuse gastric cancer. J Pathol.

[36] Schuetz JM, Leach S, Kaurah P, Jeyes J, Butterfield Y, Huntsman D (2012). Catenin family genes are not commonly mutated in hereditary diffuse gastric cancer. Cancer Epidemiol Biomarkers Prev.

[37] Guerra J, Pinto C, Pinto P, Pinheiro M, Santos C, Peixoto A (2023). Frequency of CDH1, CTNNA1 and CTNND1 germline variants in families with diffuse and mixed gastric cancer. Cancers.

[38] Herzig M, Savarese F, Novatchkova M, Semb H, Christofori G (2007). Tumor progression induced by the loss of E-cadherin independent of β-catenin/Tcf-mediated Wnt signaling. Oncogene.

[39] Hong JY, Oh I-H, McCrea PD (2016). Phosphorylation and isoform use in p120-catenin during development and tumorigenesis. Biochim Biophys Acta.

[40] Thoreson M, Anastasiadis P, Daniel J, Ireton R, Wheelock M, Johnson K (2000). Selective uncoupling of P120 from E-cadherin disrupts strong adhesion. J Cell Biol.

[41] Wehrendt DP, Carmona F, González Wusener AE, González Á, Martínez JML, Arregui CO (2016). P120-catenin regulates early trafficking stages of the N-cadherin precursor complex. PLoS ONE.

[42] Yu J, Miao Y, Xu H, Liu Y, Jiang G (2012). N-terminal 1–54 amino acid sequence and armadillo repeat domain are indispensable for P120-catenin isoform 1A in regulating E-Cadherin. PLoS ONE.

[43] Yanagisawa M, Kaverina IN, Wang A, Fujita Y, Reynolds AB, Anastasiadis PZ (2004). A novel interaction between kinesin and p120 modulates p120 localization and function. J Biol Chem.

[44] Guilford P, Hopkins J, Harraway J, McLeod M, McLeod N, Harawira P (1998). E-cadherin germline mutations in familial gastric cancer. Nature.

[45] Pocurull A, Herrera-Pariente C, Carballal S, Llach J, Sánchez A, Carot L (2021). Clinical, molecular and genetic characteristics of early onset gastric cancer: analysis of a large multicenter study. Cancers.

[46] Corso G, Carvalho J, Marrelli D, Vindigni C, Carvalho B, Seruca R (2013). Somatic mutations and deletions of the E-cadherin gene predict poor survival of patients With gastric cancer. J Clin Oncol.

[47] Anastasiadis PZ, Reynolds AB (2001). Regulation of Rho GTPases by p120-catenin. Curr Opin Cell Biol.

