# Supplementary material for: CTNND1 is involved in germline predisposition to early-onset gastric cancer by affecting cell-to-cell interactions
Source: Gastric Cancer. 2024 May 25;27(4):747–59. doi: 10.1007/s10120-024-01504-7 (PMC11193828; doi:10.1007/s10120-024-01504-7)
Supplement: Supplementary file 1 — Supplementary file1 (PDF 24847 KB) [file 10120_2024_1504_MOESM1_ESM.pdf]

SUPPLEMENTARY MATERIAL

SUPPLEMENTARY MATERIALS AND METHODS

Patients

Immunohistochemistry and loss of heterozygosity

Immunostainings of p120ctn and E-cadherin were performed when tumour samples were available. Histological 2-µm sections from stomach tumor from patients and normal mucosa from controls were used. After deparaffination, antigen retrieval was done with citrate buffer 10 mM, and tissue was permeabilized with 1% Triton X-100. Peroxidase activity was blocked with 3% hydrogen peroxide. Sections were treated for one hour with Dako serum-free protein blocker (Agilent, Santa Clara, CA), incubated overnight with monoclonal rabbit anti-p120ctn or anti-E-cadherin antibodies at 1:300 dilution for 1 hour with goat anti-rabbit secondary antibody at RT (Dako REAL EnVision HRP Rabbit; Agilent). Sections were revealed with diaminobenzidine for 90 seconds (Agilent), stained with hematoxylin, and mounted. For visualization, 200x and 400x objectives were used. Loss of heterozygosity (LOH) was also evaluated comparing Sanger sequencing results of germline and tumor DNA from the same patient. LOH could be performed only when tumor sample was available.

Variant identification

Gene panel sequencing

Twenty-four candidate genes were included in the gene panel according to results in the discovery cohort (*ARID4A*, *BCL6B*, *CTNND1*, *DACT2*, *FAT1*, *FAT2*, *FAT4*, *HBP1*, *KAT5*, *LIG3*, *MAD1L1*, *PHF2*, *RAD23A*, *TLR2*, *TLR5*, *UNG*, *APC*, *ATM*, *BAP1*, *ERCC2*, *FANCA*, *POT1*, *SDHC* and *RNF43*). Also, other genes were added due to their previous involvement in hereditary GC (*BRCA1*, *BRCA2*, *CTNNA1*, *PALB2*, *CDH1*, *MLH1*, *MSH2*, *MSH6*, *STK11*, *SMAD4*, *TP53*, *MUTYH, BMPR1A* and *RAD51C*).

Gene-panel sequencing was completed using the Illumina Miseq platform (Illumina, San Diego, USA). Library quality control was achieved with Bioanalyzer 2000 (Agilent, Santa Clara, CA, USA). Samples were indexed with adapters containing different barcodes and pooled together. Then, massively parallel sequencing was accomplished using an AmpliSeq DNA panel of 1308 amplicons divided into two pools, with an amplicon length of 128-275 bp (Illumina, San Diego, USA).

Cellular model development for variant characterization

Generation of *CTNND1* loss-of-function model

A single guide RNA (sgRNA) against exon 7 of *CTNND1* was designed, as it is the first common exon among the 40 transcripts described for this gene. Benchling CRISPR Guide design tool (https://benchling.com/) was used to this end. Bottom and top strands of sgRNA were

purchased from IDT (Coralville, IA) and cloned into the BbsI site of the PX458 plasmid (Cat No. 48138, Addgene). The plasmid was transiently transfected into NCI-N87 and hTERT RPE-1 cells using Lipofectamine 3000 reagent (ThermoFisher, Waltham, MA). After 48h, GFP-positive cells were selected by FACS-sorting. Then, they were seeded into 96-well plates at a density of one cell per well. For clone isolation, NCI-N87 cells required conditioned media. This enriched media was prepared by seeding NCI-N87 cells at a density of 1 million cells per 100 mm plate and collecting the supernatant after 48 hours. After one month, several clones were analyzed for *CTNND1* gene editing. DNA was extracted and gene editing was evaluated by Sanger sequencing (Eurofins Genomics). Protein level was also evaluated by Western blot. Primers used are listed in Supplemental Table 1.

Site-directed mutagenesis

The open reading frame of *CTNND1*, encoding the longest protein and canonical transcript (isoform 1ABC), was purchased from IDT (IDT HiFi Gblocks). DYK-tag (DYKDDDDK) was added before the codon stop for screening and detection purposes. The double-stranded DNA coding sequence was cloned into the pUC19 carrier vector (Cat No. 18265017, Invitrogen, Waltham, MA) flanked by EcoRI and AgeI restriction sites. Once *CTNND1*-pUC19 was generated, site-directed mutagenesis was carried out using Q5 Site-directed Mutagenesis kit (NEB, Ipswich, MA) according to manufacturer's instructions. Back-to-back primers were designed using the NEBaseChanger tool and purchased at IDT (Coralville, IA). Primers are listed in Supplemental Table 1. Variant insertion was verified by Sanger sequencing.

Variant reintroduction into the *CTNND1* knock-out model

Each of the *CTNND1* variants was individually inserted into the inducible lentiviral plasmid pLVXTetOne-Puro (Cat No. 631847, TakaraBio, Kusatsu, Japan) using EcoRI and AgeI enzymes, generating a stable and reproducible *CTNND1* expression system. Four different pLVXTetOne-Puro-*CTNND1* plasmids were generated, including the three candidate *CTNND1* variants, as well as the wild-type sequence to rescue the original phenotype.

Lentiviral preparation and transduction

Each pLVXTetOne-Puro-*CTNND1* plasmid was packaged into lentiviral particles using HEK293T as a host cell line and the CalPhos Mammalian Transfection Kit (TakaraBio, Kusatsu, Japan). Once cells were seeded, they were transfected with the lentiviral vectors VSVG2, psPAX2 and pLVXTetOne-Puro-*CTNND1* (either wildtype or mutated version). The supernatant was recovered at two different time points (24 and 48 hours after transfection), mixed, filtered through a 0.45 µm filter (Millipore) and concentrated in a 10% sucrose cushion by centrifugation (20,000 xg, 3 hours, 4ºC). Then, the lentiviral particles were resuspended in 2.5 mL of RPMI-1640 medium and prepared for infection.

CTNND1 knockout cells (NCI KO10 and RPE KO7) were seeded at a density of 600,000 cells/well and 200,000 cells/well in 6-well plates, respectively. After two days, cell transduction

was performed with different viral dilutions by centrifugation (1600 xg, 2h, 32ºC) in combination with 8 µg/mL of polybrene. Then, the supernatant was replaced with the appropriate cell media (RPMI-1640 or DMEM), and infected cells were enriched by puromycin selection (1 µg/mL or 20 µg/mL, respectively).

Optimization of the TetOne system

The dose-dependent response to doxycycline of infected cells was assessed. A final dose of 100 ng/mL doxycycline was selected to successfully induce gene expression of each of the introduced *CTNND1* variants. Cells were induced two days before cell seeding, and doxycycline treatment was refreshed every two days.

Protein extraction and Western blot

Cells were scrapped, pelleted and then lysed using RIPA buffer solution (Sigma-Aldrich, MA, USA) supplemented with cOmplete Protease Inhibitor Cocktail and PhosSTOP (Roche, Basel, Switzerland). Protein extracts were quantified with the Pierce BCA Protein Assay kit (ThermoFisher, Waltham, MA). For NCI-N87 and hTERT-RPE models, 40 µg and 20 µg of total protein extracts were used, respectively. Proteins were separated on NuPAGE 4-12% Bis-Tris gels according to the manufacturer's protocol (ThermoFisher, Waltham, MA). Then, proteins were transferred into PVDF membranes (Millipore, Bedford, MA). Blots were probed with anti-CTNND1 (clone D7S2M, Cat No. 59854, Cell Signaling) and anti-GAPDH (clone 14C10, Cat No. 2118, Cell signaling) primary antibodies. Then, incubation with 800CW anti-mouse (Cat No 926-32210, LI-COR, Lincoln, NE) or 680RD anti-rabbit (Cat No 926-68071, LI-COR, Lincoln, NE) IRDye secondary antibodies was done, depending on the host species of the primary antibody. Protein detection was carried out using the Odyssey Imaging System (LI-COR, Lincoln, NE) and analyzed with Image Studio 4.0 software.

Detachment assay

Cells were seeded at 140,000 cells/well and 50,000 cells/well (for NCI-N87 and hTERT-RPE cell models, respectively) in 48-well plates. Next day, cells were washed with PBS and treated with trypsin pre-warmed at 37 ºC at different timepoints (0, 4, 8, and 12 minutes for NCI-N87 and 0, 3 and 6 minutes for hTERT-RPE cells). Then, cells were blocked with medium supplemented with 10% foetal bovine serum. Immediately, detached cells were removed carefully. A control well without trypsin was included in each experiment. Cells were fixed with cold methanol during 10 minutes at 4ºC and stained with 0.5% crystal violet solution during 15 minutes at RT. Excess crystal violet was washed out with distilled water, and plates were let to air-dry overnight. Crystal violet was diluted by adding 400 µl of methanol per well and absorbance at 570 nm was measured. For variant characterization, cell detachment was assessed only at the critical timepoint.

Uncropped Western blot

Uncropped Western Blot
Figure 1A and Figure 2A

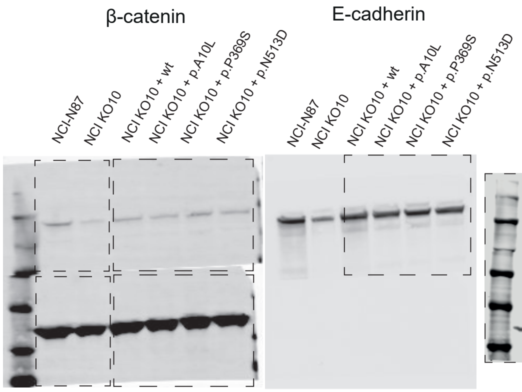

Uncropped Western Blot
Supplemental Figure 3B

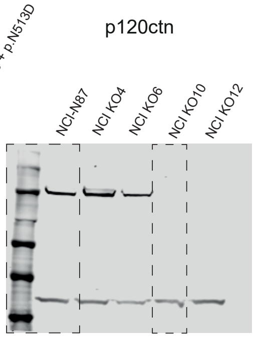

Uncropped Western Blot
Supplemental Figure 3D

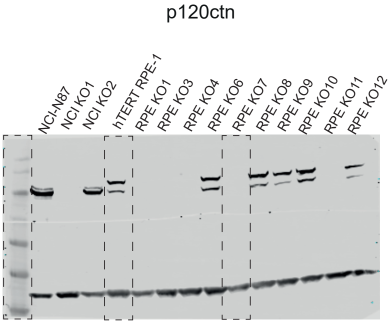

Uncropped Western Blot
Supplemental Figure 3G
(upper panel)

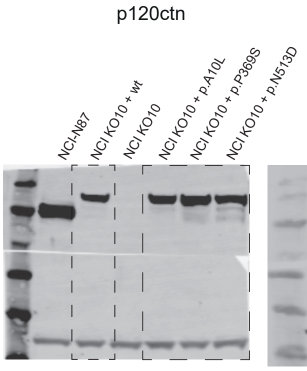

Uncropped Western Blot
Supplemental Figure 3G
(down panel)

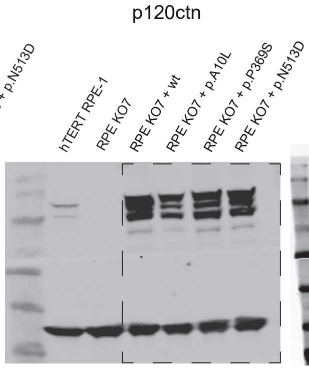

Uncropped Western Blot
Supplemental Figure 4A
and Figure 5A

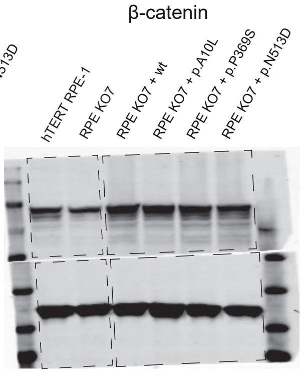

SUPPLEMENTARY TABLES

Supplementary Table S1. List of primers used in this study.

	Forward (5'-3')	Reverse (5'-3')
**Validation of genetic variants in discovery and replication cohorts**		
CTNND1 (c.28_29delinsCT)	TCTCTCCCTCCTTCTCCTTC	CGGGTCAGCTTCTCAAACT
CTNND1 (c.1105C>T)	GTGAGGAGGTGCCATCGGAT	CTTGAGCTTCCGCACGTCAG
CTNND1 (c.1537A>G)	TTGTCTCTTCCAGGAACCCT	CTGTTACCTAAGGCAGCCAG
**CTNND1 knock-out generation**		
CTNND1 sgRNA	caccGTCTGGCTGTCTCCAATTAGG	aaacCCTAATTGGAGACAGCCAGAC
CTNND1 knock-out validation	TGGGAATGTCCAGGAAGCTA	CTCCAAGGTGCACTTCCTTT
**Site-directed mutagenesis and cloning**		
Cloning AgeI in pUC19	AATTCCCGTATACACCGGTGCCG	GATCCGGCACCGGTGTATACGGG
CTNND1 (c.28_29delinsCT)	GGAGTCGACCCTCAGCATCTTGG	ACCTCTGAGTCGTCCATG
CTNND1 (c.1105C>T)	GCCAGAGCTGTCAGAGGTGAT	TGTCTCCAATTAGGAGGTGG
CTNND1 (c.1537A>G)	GCGGGAACCTGATGAAGACTG	TCCCAACCAGAATGAGGAAT
c.28_29delinsCT validation	GCACAGATGCGTAAGGAGAA	TTGGCATCTTGTGGTGAGAC
c.1105C>T validation	GTGAGGAGGTGCCATCGGAT	CTTGAGCTTCCGCACGTCAG
c.1537A>G validation	AAAACTGTGATGGTGTGCCT	CTGTCTGAATCCTTCTGCCC

Supplementary Table S2. Overview of variant details

Gene	Genomic coordinates (GRCh38)	Transcript (MANE selected)	cDNA	aa change	Pathogenicity predictors	gnomAD frequence (v.3.1.2)	Functional domain
CTNND1	chr11:57791506_57791507	NM_001085458.2	c.28_29delinsCT	p.(Ala10Leu)	6	No match	coiled-coil domain
CTNND1	chr11:57801881	NM_001085458.2	c.1105C>T	p.(Pro369Ser)	3	1.31e-5	ARM1
CTNND1	chr11:57803737	NM_001085458.2	c.1537A>G	p.(Asn513Asp)	3	3.62e-4	ARM4

SUPPLEMENTARY FIGURES

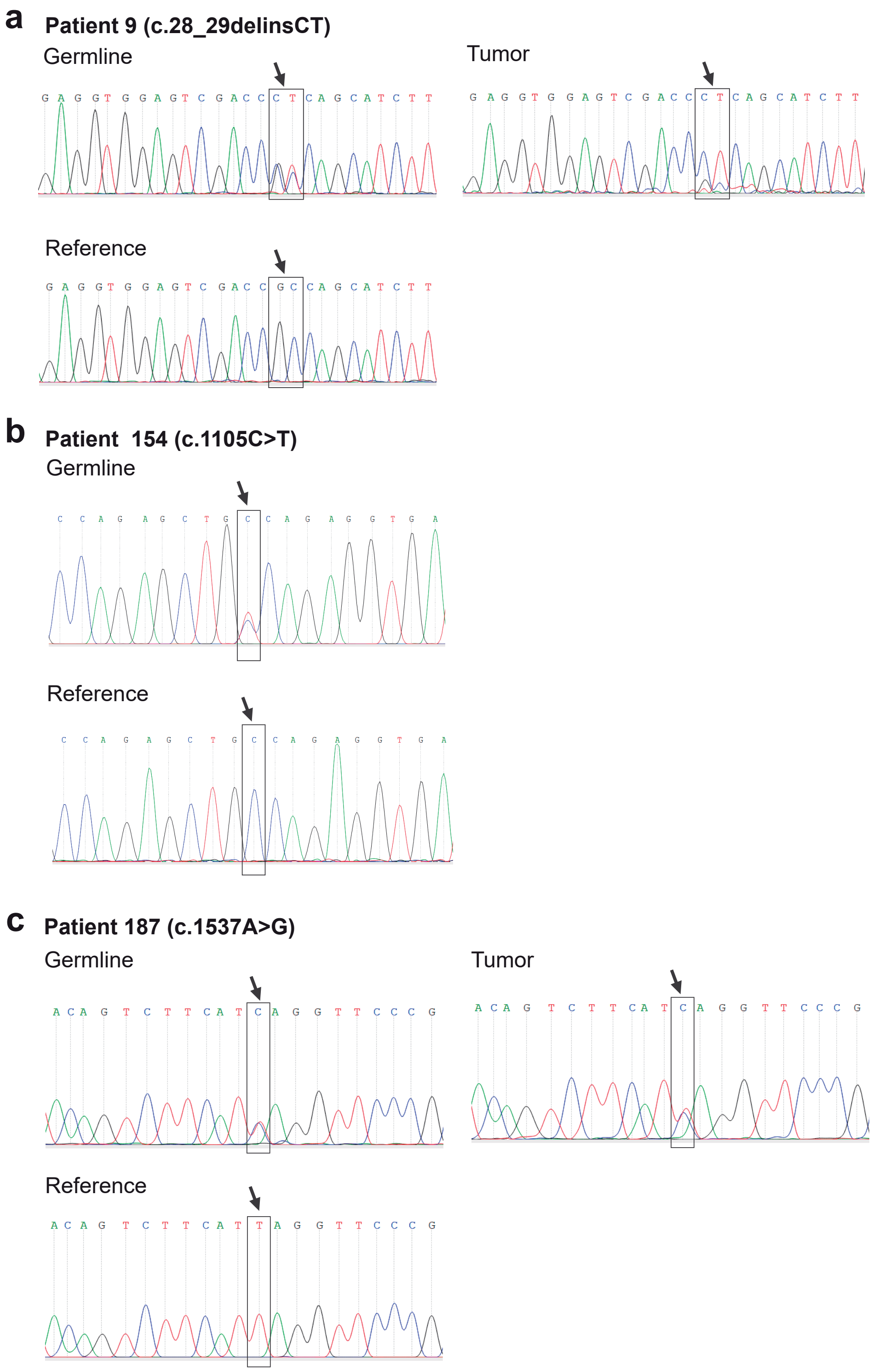

Supplementary Fig.S1 Variant validation and loss of heterozygosity analysis in *CTNND1* variant carriers. **a-c** Sanger sequencing confirmed the presence of the three *CTNND1* genetic variants in germline DNA (c.28_29delinsCT (p.Ala10Leu), c.1105C>T (p.Pro369Ser) and c.1537A>G (p.Asn513Asp)). Somatic Sanger sequencing in those available tumor samples. In patient 9 (c.28_29delinsCT), a decrease of the wild-type allele was found in the tumor

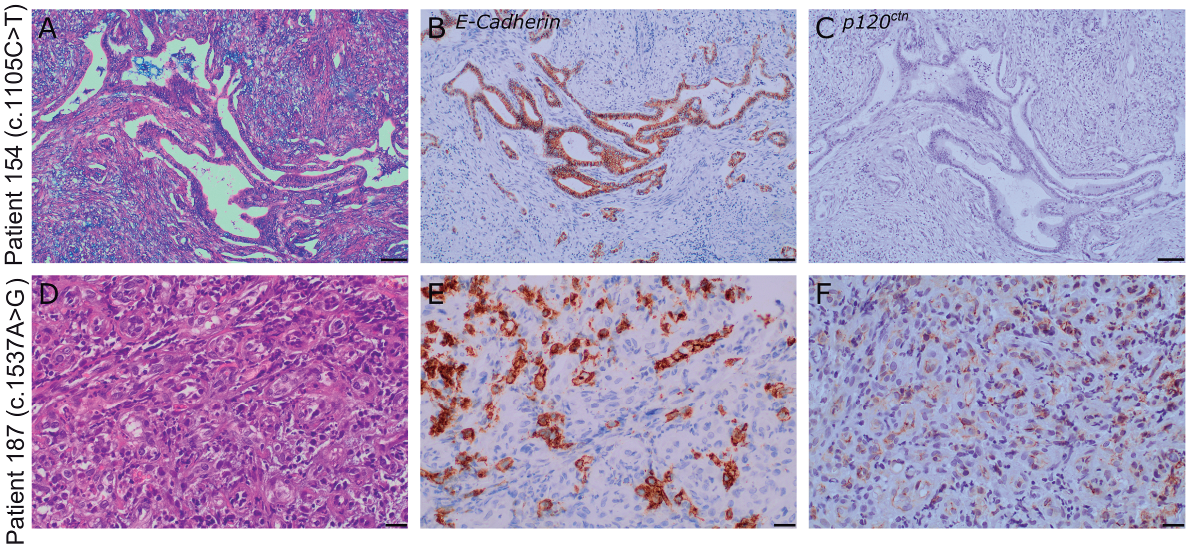

Supplementary Fig.S2 Immunostains of E-cadherin and p120ctn. **a-c** Tubular (intestinal) adenocarcinoma positive for E-Cadherin and negative for p120ctn in c.1105C>T p.(Pro369Ser) variant carrier, scale bar=50 µm. **d-f** Poorly cohesive (diffuse) carcinoma with preserved E-cadherin and p120ctn staining in c.1537A>G (p.Asn513Asp) variant carrier. Some tumor cells show focal and weak membrane staining, scale bar=20 µm

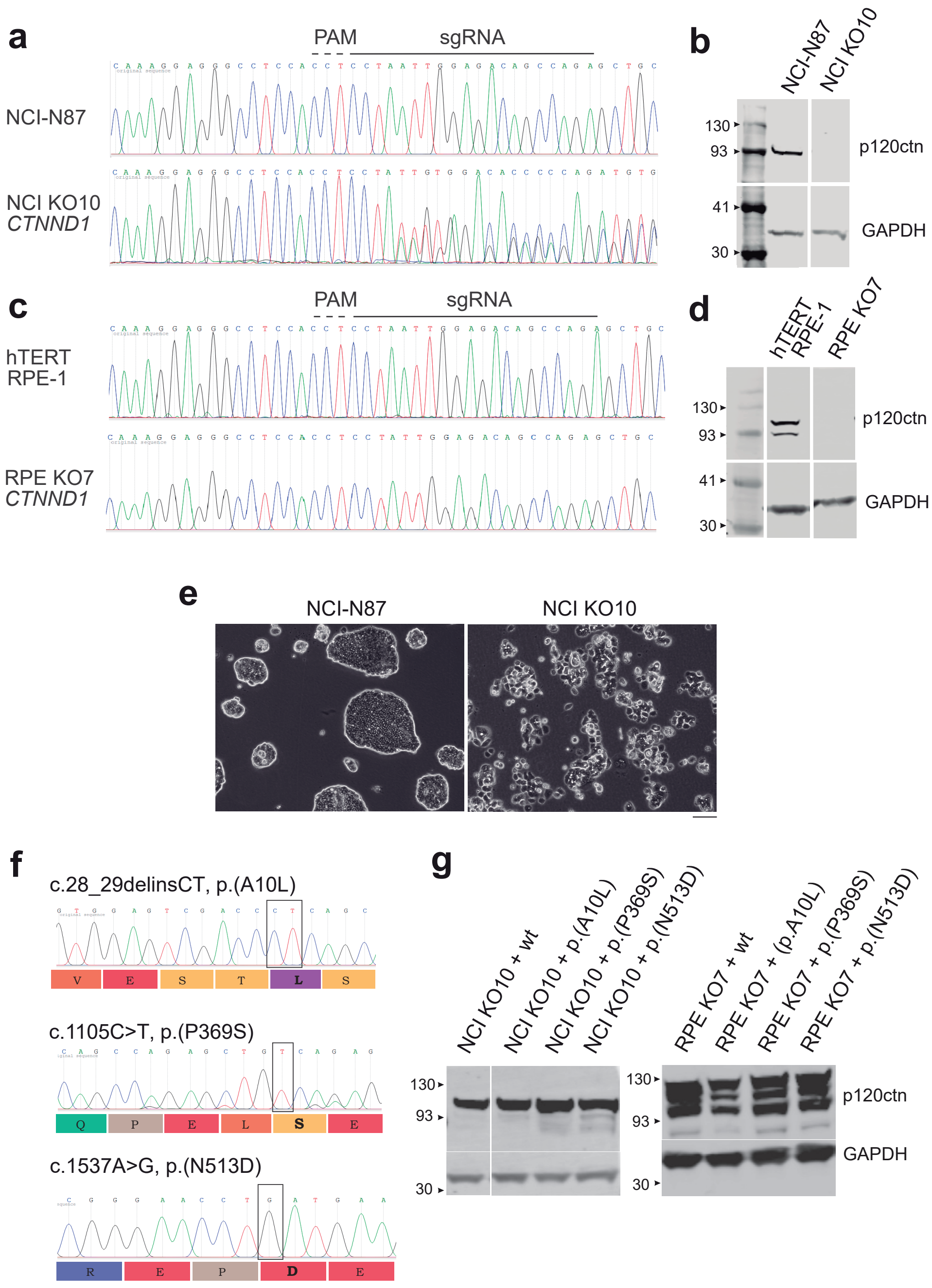

Supplementary Fig.S3 Validation of the CRISPR/Cas9-driven *CTNND1* editing and *CTNND1* expression. **a,c** Sanger sequencing confirmed an insertion and deletion of 1 bp in exon 7 in NCI KO10 model, as well as a homozygous deletion of 1 bp in exon 7 in RPE KO7 model. The target sgRNA is indicated by a line, and the corresponding PAM sequence with a dashed line. **b,d** p120ctn protein levels were evaluated by Western blot, confirming the complete inactivation of the gene in both models. Representative blots of n=3. GAPDH was used as an internal control. **e** Evident morphological changes in NCI-N87 cells were observed after *CTNND1* inactivation. Scale bar=200µm. **f** Site-directed mutagenesis of pLVX-TetOne-Puro-CTNND1 constructs. Changes in the nucleotide sequence are marked, and the altered amino acid is highlighted in bold. **g** p120ctn re-expression was confirmed by Western blot in both knock-out models

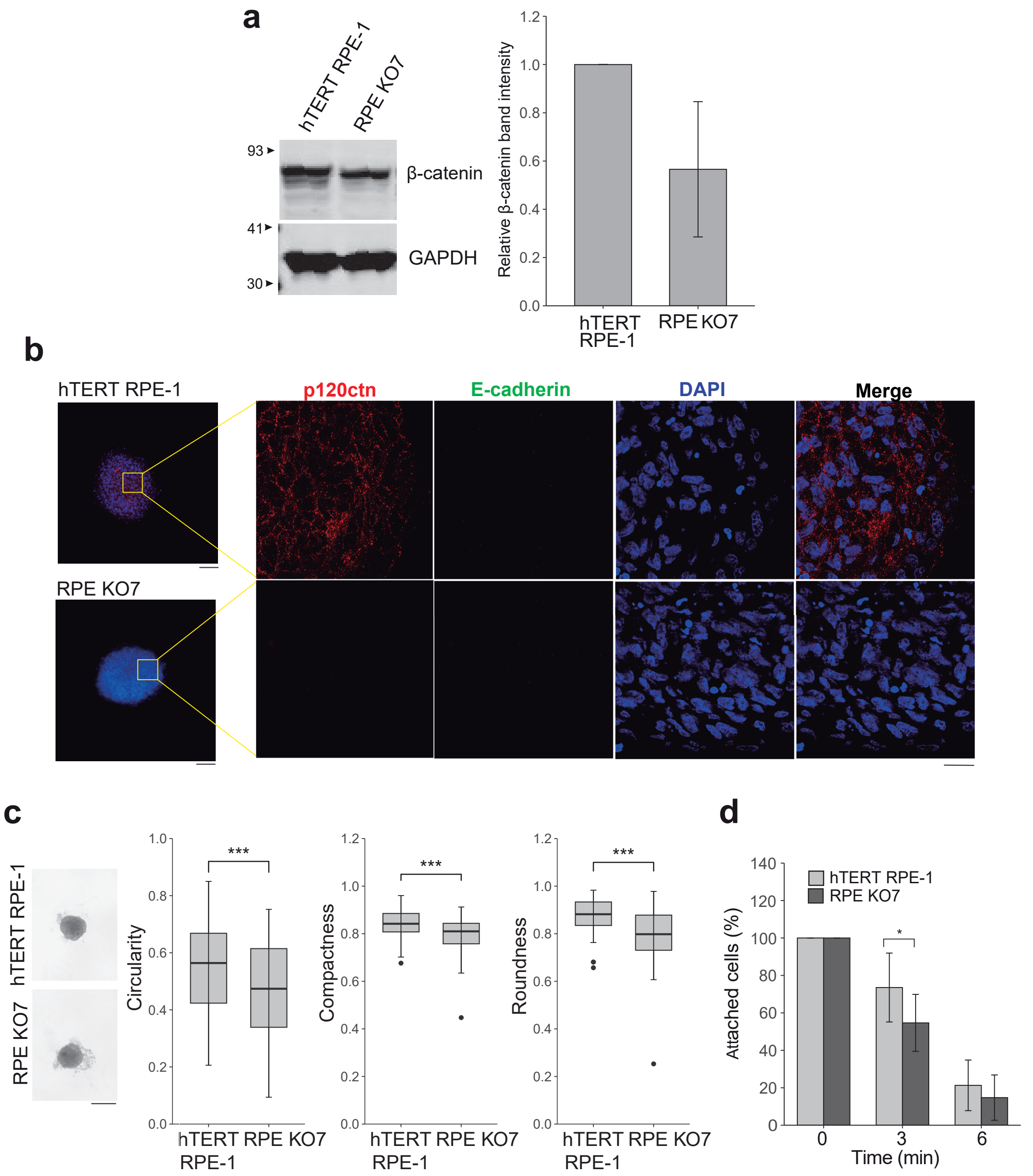

Supplementary Fig.S4 Functional characterization of the *CTNND1*-depleted hTERT RPE-1 cell model. **a** Quantification of relative protein levels of β-catenin by Western blot. GAPDH was used as internal control. Representative blot of n=3 (mean±SD; Welch's t-test). **b** Immunofluorescent staining of spheroids (hTERT RPE-1 and RPE KO7) using p120ctn and E-cadherin antibodies. hTERT RPE-1 cells showed a dotted, vesicular-like p120ctn expression pattern. CRISPR-mediated *CTNND1* editing resulted in p120ctn complete depletion. The lack of E-cadherin expression in hTERT RPE-1 cell line was also confirmed. Wide-field microscopy images (left panel) were captured using a 10x objective (Scale bar=100 µm). A small area is shown at higher magnification (right panel, 63x oil objective, scale bar=20 µm). **c** hTERT RPE-1 and RPE KO7 spheroids. Scale bar=200 µm. All shape descriptors were affected after p120ctn deletion. Spheroids were generated at least in quintuplicate and the experiment was repeated 5 times (n=5; mean±SD; Welch's t-test, ***P<.001). **d** Detachment time-course assay. Data represents the percentage of remaining attached cells after being treated with trypsin at different timepoints (n=9, mean±SD; Welch's t-test, P<.05)

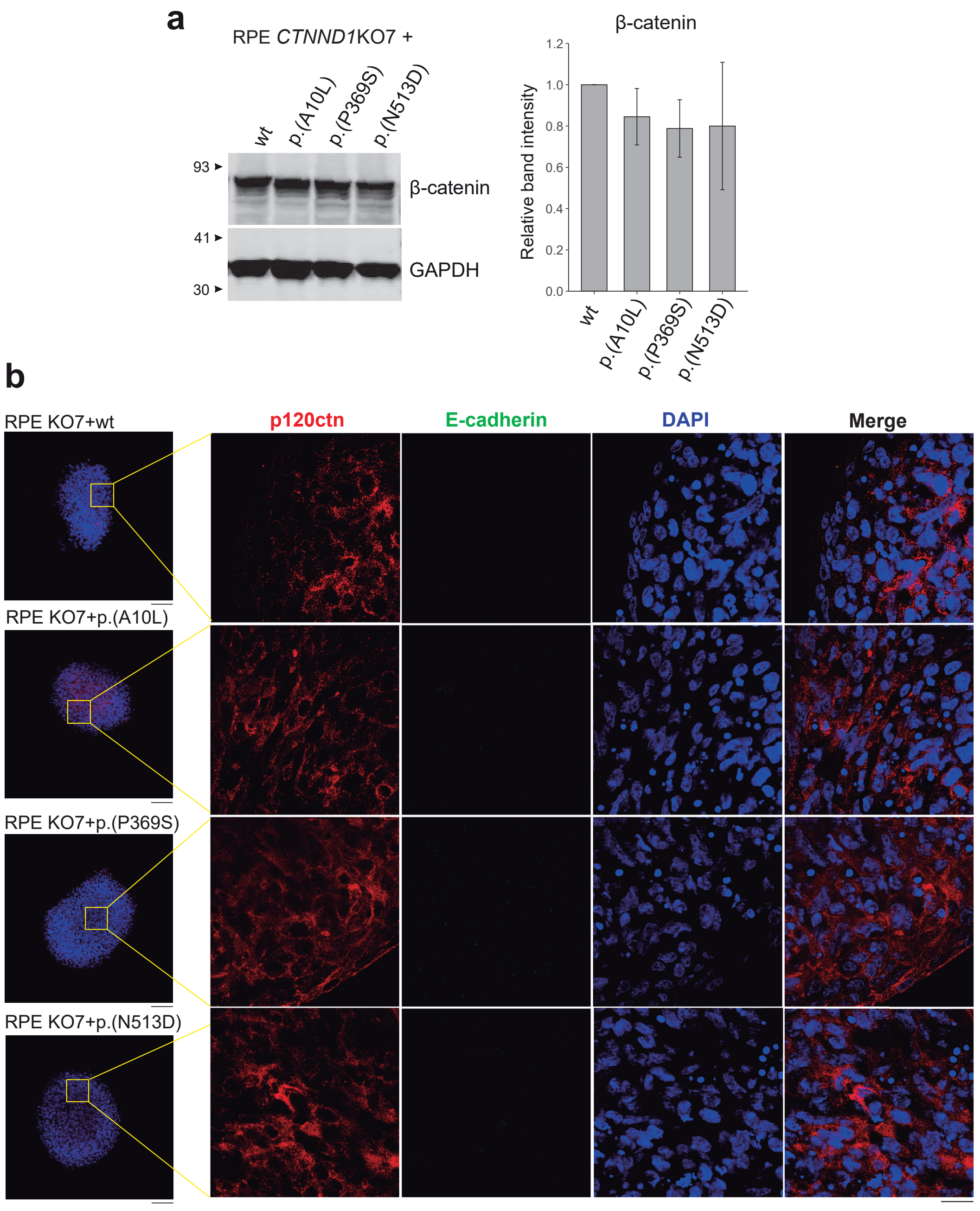

Supplementary Fig.S5 Functional characterization of the RPE model expressing *CTNND1* variants: Wnt pathway and catenin localization. **a** Quantification of relative protein levels of β-catenin by Western blot. GAPDH was used as internal control. Representative blot of n=3 (mean±SD; analysis of variance with LSD post hoc test). **b** Immunofluorescent staining of spheroids. Wild-type and p120ctn variants showed a dotted, vesicular staining pattern, suggesting mislocalization and internalization. No E-cadherin expression was found. Wide-field microscopy images (left panel) were captured using a 10x objective (Scale bar=100 µm). A small area is shown at higher magnification (right panel, 63x oil objective, scale bar=20 µm). Protein annotation is in its short form

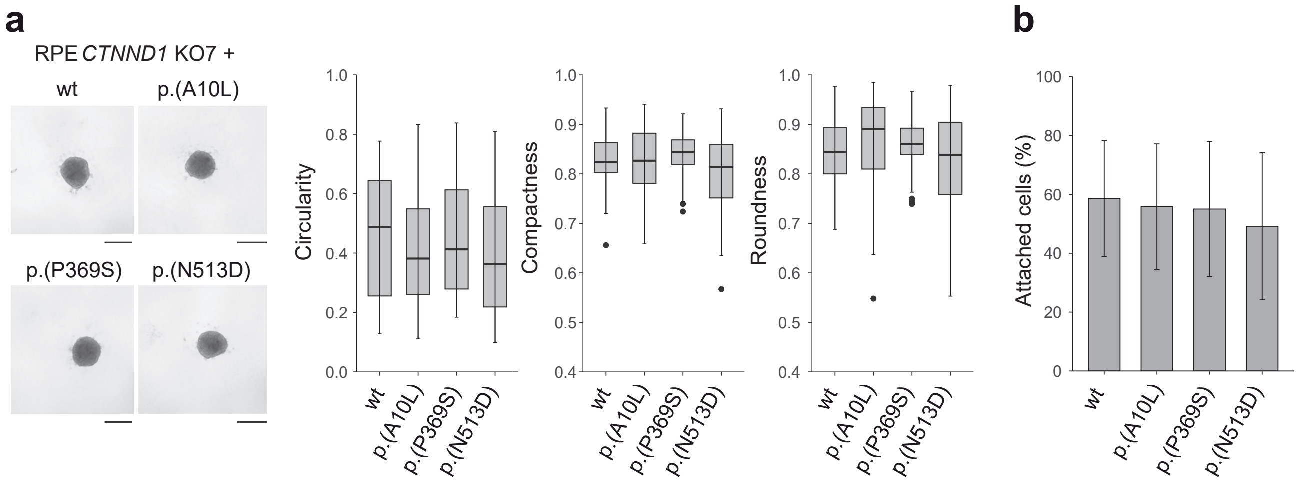

Supplementary Fig.S6 Functional characterization of the RPE model expressing *CTNND1* variants: spheroid modelling and detachment assay. **a** Representative picture of spheroids expressing each *CTNND1* variant or its wild-type counterpart. Circularity, compactness and roundness index are represented. Spheroids were generated at least in quintuplicate and the experiment was repeated 6 times (n=6; mean±SD; analysis of variance with LSD post hoc test). **b** Detachment assay. Data represents the percentage of remaining attached cells after being treated with trypsin for 3 minutes (n=10, mean±SD; analysis of variance with LSD post hoc test). Protein annotation is in its short form
